# Normoalbuminuric kidney impairment in patients with T1DM: insights from annals initiative

**DOI:** 10.1186/s13098-018-0361-2

**Published:** 2018-07-31

**Authors:** Olga Lamacchia, Francesca Viazzi, Paola Fioretto, Antonio Mirijello, Carlo Giorda, Antonio Ceriello, Giuseppina Russo, Pietro Guida, Roberto Pontremoli, Salvatore De Cosmo

**Affiliations:** 1 Unit of Endocrinology and Metabolic Diseases, Department of Surgical and Medical Sciences, University of Foggia, Foggia, Italy; 2 Department of Internal Medicine, University of Genoa and Policlinico San Martino, Genoa, Italy; 3 Department of Medicine, University of Padova, Padova, Italy; 4 Department of Medical Sciences, Scientific Institute “Casa Sollievo della Sofferenza”, San Giovanni Rotondo, FG Italy; 5 Diabetes and Metabolism Unit ASL Turin 5, Chieri, Italy; 6 Institut d’Investigacions Biomèdiques August Pii Sunyer (IDIBAPS) and Centro de Investigación Biomédicaen Red de Diabetes y Enfermedades Metabólicas Asociadas (CIBERDEM), Barcelona, Spain; 7 U.O. Diabetologia e Malattie Metaboliche, Multimedica IRCCS, Sesto San Giovanni, Milan, Italy; 8 Department of Clinical and Experimental Medicine, University of Messina, Messina, Italy; 9 Associazione Medici Diabetologi, Rome, Italy

**Keywords:** Albuminuria, Chronic kidney disease, Type 1 diabetes mellitus, Cardiovascular disease

## Abstract

**Background:**

We described, in a large sample of patients with type 1 diabetes mellitus (T1DM) and GFR ≤ 60 mL/min/1.73 m^2^ (with or without albuminuria), the differences in the clinical features associated with the two different chronic kidney disease phenotypes and we investigated, in a subset of patients, the modulating role of albuminuria on kidney disease progression.

**Methods:**

Clinical data of 1395 patients with T1DM were extracted from electronic medical records.

**Results:**

Albuminuria was detected in 676 (48.5%) patients, with the remaining 719 (51.5%) patients having normoalbuminuric renal impairment. Those with albuminuria showed an evident worse cardiovascular risk profile as compared to patients with normoalbuminuria. A subgroup of 582 patients was followed up over a 4-year period. One hundred and twenty five patients (21.5%) showed a loss of eGFR > 30%. The proportion of patients reaching the renal outcome was significantly higher among those with baseline albuminuria as compared to patients with normoalbuminuria (P < 0.0001). At the multivariate logistic analysis microalbuminuria, macroalbuminuria and proliferative retinopathy were the only parameters independently associated to eGFR reduction.

**Conclusions:**

The proportion of T1DM patients with normoalbuminuria renal impairment is high (about 50%). These patients have a slower eGFR decline as compared to that observed in patients with albuminuria renal impairment.

**Electronic supplementary material:**

The online version of this article (10.1186/s13098-018-0361-2) contains supplementary material, which is available to authorized users.

## Background

Diabetic kidney disease (DKD), an important complication of diabetes mellitus, is the leading cause of kidney failure in the western word and it is also associated with increased cardiovascular morbidity and mortality. Albuminuria and reduced glomerular filtration rate (GFR) are the key features of DKD. Traditionally, the earliest clinical manifestation of DKD has been the detection of persistent microalbuminuria [1]. Early studies suggested that GFR starts to decrease only when albuminuria reaches the macroalbuminuric range [2]. This conventional paradigm of kidney disease in people with diabetes has been recently challenged. Changes in albuminuria and GFR are being increasingly recognized as complementary rather than obligatory manifestations of DKD. Furthermore, albuminuria and loss of GFR seem to be associated with distinct clusters of specific risk factors. Several studies investigating the prevalence of both normoalbuminuric and albuminuric chronic kidney disease (CKD) have been performed in type 2 diabetes mellitus (T2DM) [3–7]. Limited information is available concerning heterogeneity of renal features in patients with type 1 diabetes mellitus (T1DM) and this is largely resulting from studies conducted in a few cohorts of patients with GFR ≤ 60 mL/min/1.73 m^2^ [8–10]. Recently, Penno et al. [10] have recently assessed the prevalence and correlates of different CKD phenotypes in a cohort of 777 patients with T1DM (of which 29 with eGFR < 60 mL/min/1.73 m^2^). They found that normoalbuminuric CKD phenotype is present in a significant proportion of the T1DM, a finding supporting two distinct pathways, with and without albuminuria, to advanced kidney disease.

We therefore analyzed a large sample of patients with T1DM and GFR ≤ 60 mL/min/1.73 m^2^ (with or without albuminuria) attending diabetes clinics and representative of current clinical practice for diabetes care in Italy to describe the differences in the clinical features associated with the two different CKD phenotypes (i.e. with and without albuminuria) and to investigate, in a subset of patients, the modulating role of albuminuria on kidney disease progression.

## Methods

In this report, we show the results of an analysis performed using the data set of the electronic medical records collected on a large sample of patients with a diagnosis of T1DM (according to American Diabetes Association 2003 criteria), who attended 295 diabetes clinics in Italy between 1 January 2004, and 31 December 2011. Approximately, one-third of all the Italian diabetes clinics were involved in this study, evenly distributed throughout the country and therefore indicative of the clinical practice. For the purpose of the analysis, we considered only patients who were aged 18 years or older, with at least 1 outpatient measurement of serum creatinine and albuminuria and with a GFR ≤ 60 mL/min/1.73 m^2^. A total of 1395 patients (654 males and 741 females) constituted the study population (Additional file 1: Fig. S1).

The database derives from the Italian Association of Clinical Diabetologists (Associazione Medici Diabetologi—AMD) initiative designed to monitor diabetes care and quality of care as previously reported [11–13]. All participating clinics used an electronic clinical record system, and a software specifically developed to extract information. Data from all participating clinics were collected anonymously and were centrally analyzed [11–13]. The results were internally approved by the AMD Annals scientific committee. The core data-set included measures and monitoring of glycated hemoglobin (HbA1c), total cholesterol, low-density lipoprotein cholesterol (LDL-c) or high density lipoprotein cholesterol (HDL-c), and triglycerides. The use of specific classes of drugs was also evaluated. Kidney function was assessed by serum creatinine, measured by modified Jaffè method, and urinary albumin excretion measurements (UAE). Glomerular filtration rate (GFR) was estimated for each patient by using the CKD epidemiology collaboration formula derived by serum creatinine values [14]. Increased UAE was diagnosed as (1) microalbuminuria if urinary albumin concentration was > 30 and ≤ 300 mg/L, or if UAE rate was > 20 and ≤ 200 μg/min, or if urinary albumin-to-creatinine ratio was > 2.5 mg/mmol in men and > 3.5 mg/mmol in women and ≤ 30 mg/mmol in both gender and (2) macroalbuminuria if urinary albumin concentration was > 300 mg/L, or if UAE rate was > 200 μg/min, or if albumin-to-creatinine ratio was > 30 mg/mmol in both gender. Urine albumin excretion was evaluated by means of urine albumin concentration in the majority of study patients 1020 (i.e. 73%) and by albumin to creatinine ratio (spot morning samples) in a smaller number of patients 344 (25%). Timed urine collection (24 h) was used in a minority of patients 31 (2%).

Albuminuria indicates patients with either microalbuminuria or macroalbuminuria. DKD was defined as diabetes with albuminuria and/or low GFR (≤ 60 mL/min/1.73 m^2^). Information on the presence of diabetic retinopathy, background (BR), or proliferative (PR), was also available. At each participating center, all patients underwent physical examination and blood pressure (BP) measurements according to a standardized protocol. BP was measured with the patient in the sitting position after a 5-min rest, with a mercury sphygmomanometer.

For the cross-sectional analyses, we extracted data from the most recent patients’ visit complete of eGFR measurement and albuminuria evaluation. To obtain the 4-year evaluations, we selected for each patient the last 4-year period that included eGFR and albuminuria status at baseline and a re-evaluation of eGFR 4 years later. As a consequence of selection criteria, the baseline included a visit up to the year 2008 and the follow-up up to 2011 (range of visits 48 ± 6 months selecting that closest to 48 month). For patients without a re-evaluation at 4-year, the last visit up to the year 2008 was considered for the cross-sectional study (the same period of baseline visit for the longitudinal population).

## Statistical analysis

Data are given as mean value ± standard deviation or median (interquartile range), and categorical variables are described as frequencies and percentages. Logistic regression analysis was used to evaluate variables associated to albuminuria. Predictors of eGFR reduction > 30% [15] than baseline were evaluated with a logistic model adjusting for baseline eGFR. A multivariate model was built selecting variables associated to the outcome with a P value < 0.05 adjusting for age and gender. Multivariate analysis was performed as complete-case analysis including patients with all data available. The odds ratios (ORs) were used to estimate the degree of association with outcome. Assuming linearity of eGFR reduction over time, its slope was taken as a measure of disease progression rate. For each patients, we calculated the regression coefficient (slope) of linear regression between eGFR value and the exact time in years from the first evaluation including all measurements from baseline to the 4-year visit. The analyses were made using STATA software, Version 14 (StataCorp, College Station, TX). P values of < 0.05 were considered statistically significant.

## Results

The main clinical features of the study population (n = 1395) at baseline, as a whole and grouped by the presence/absence of albuminuria, are summarized in Table 1. Overall, the mean age of patients was 63 ± 14 years, 46.9% were male and mean duration of diabetes was 27 ± 14 years. Taking into account the presence of CKD, glycemic and blood pressure controls were fairly good. A total of 986 (70.7%) and 611 (43.8%) patients were on antihypertensive and on lipid-lowering treatment, respectively. In our sample, albuminuria was detected in 676 (48.5%) patients, with the remaining 719 (51.5%) patients having normoalbuminuric renal impairment (Table 1). Those with albuminuria were more likely to be males and, on average, younger respect to patients without albuminuria. Duration of disease was comparable between patients with or without albuminuria. Albuminuria was also associated with a worse glycemic control and higher levels of serum uric acid. Furthermore, albuminuria was associated with a more atherogenic lipid profile (i.e. higher LDL cholesterol and triglycerides levels and lower HDL-c) and higher values of systolic BP (SBP) and diastolic BP (DBP) despite a greater prevalence of antidyslipidemic and antihypertensive treatment.

**Table 1:** Baseline clinical characteristics of 1395 T1DM patients with low eGFR as whole and after stratification by the presence of albuminuria

	Overall	Normoalbuminuria	Albuminuria	P
	n = 1395	n = 719	n = 676	
Male sex	654 (46.9%)	252 (35.0%)	402 (59.5%)	< 0.001
Age (years)	63 ± 14	66 ± 12	60 ± 14	< 0.001
Duration of diabetes (years)	27 ± 14	27 ± 15	27 ± 13	0.968
BMI (kg/m^2^)	26.3 ± 4.7	26.2 ± 4.5	26.5 ± 4.9	0.201
Serum creatinine (mg/dL)	1.55 ± 0.81	1.35 ± 0.52	1.78 ± 0.98	< 0.001
eGFR (mL/min/1.73 m^2^)	46 ± 12	49 ± 10	43 ± 13	< 0.001
Albuminuria	676 (48.5%)	–	–	
Serum uric acid (mg/dL)	5.7 ± 1.8	5.3 ± 1.8	6.1 ± 1.6	< 0.001
Serum uric acid in the top quintile	162 (19.0%)	60 (14.3%)	102 (23.5%)	0.001
HbA1c (%)	8.2 ± 1.6	8.0 ± 1.5	8.3 ± 1.7	< 0.001
HbA1c (mmol/mol)	66 ± 17.5	64.0 ± 16.4	67 ± 18.6	< 0.001
HbA1c ≥ 7%	1071 (77.4%)	541 (75.7%)	530 (79.2%)	0.114
HbA1c ≥ 54 mmol/mol	1071 (77.4%)	541 (75.7%)	530 (79.2%)	0.114
Total cholesterol (mg/dL)	195 ± 44	192 ± 38	198 ± 48	0.012
Triglycerides (mg/dL)	122 ± 82	110 ± 74	136 ± 89	< 0.001
Triglycerides ≥ 150 mg/dL	292 (22.4%)	113 (16.8%)	179 (28.5%)	< 0.001
HDL (mg/dL)	60 ± 19	61 ± 19	58 ± 20	0.008
HDL < 40 M < 50 F mg/dL	269 (20.8%)	133 (20.0%)	136 (21.6%)	0.491
LDL (mg/dL)	112 ± 35	109 ± 32	114 ± 38	0.027
LDL ≥ 100 mg/dL	785 (61.1%)	402 (60.4%)	383 (61.9%)	0.578
Systolic BP (mmHg)	139 ± 20	137 ± 20	141 ± 20	< 0.001
Diastolic BP (mmHg)	77 ± 10	76 ± 10	78 ± 10	0.006
Blood pressure ≥ 140/85 mmHg	659 (57.8%)	315 (53.9%)	344 (61.9%)	0.007
Non-proliferative retinopathy	279 (20.0%)	152 (21.1%)	127 (18.8%)	0.272
Proliferative retinopathy	146 (10.5%)	56 (7.8%)	90 (13.3%)	0.001
Smokers	107 (18.7%)	40 (13.5%)	67 (24.3%)	0.001
Lipid-lowering treatment	611 (43.8%)	301 (41.9%)	310 (45.9%)	0.133
Treatment with statins	579 (41.5%)	287 (39.9%)	292 (43.2%)	0.214
Treatment with fibrates	14 (1.0%)	9 (1.3%)	5 (0.7%)	0.343
Antihypertensive treatment	986 (70.7%)	453 (63.0%)	533 (78.8%)	< 0.001
Treatment with ACE-Is/ARBs	862 (61.8%)	384 (53.4%)	478 (70.7%)	< 0.001
Aspirin	339 (24.3%)	158 (22.0%)	181 (26.8%)	0.037
Insulin pump	52 (3.7%)	17 (2.4%)	35 (5.2%)	0.007

Mean ± SD or absolute frequency (percentage). Patients’ baseline missing data: duration of diabetes 72 (5.2%), BMI 212 (15.2%), serum uric acid 542 (38.9%), HbA1c 11 (0.8%), total cholesterol 73 (5.2%), triglycerides 92 (6.6%), HDL 99 (7.1%), LDL 110 (7.9%), blood pressure 255 (18.3%), smokers 823 (59.0%). Serum uric acid in the top gender-specific quintile: > 6.6 mg/dL in females and > 7.4 mg/dL in males

*eGFR* estimated glomerular filtration rate, *BMI* body mass index, *HbA1c* glycated haemoglobin, *HDL* high-density lipoprotein cholesterol, *LDL* low-density lipoprotein cholesterol, *ACE-Is* angiotensin converting enzyme-inhibitors, *ARBs* angiotensin II receptor antagonists

As expected, individuals with albuminuria were more frequently treated with angiotensin converting enzyme inhibitors (ACE-Is) or angiotensin II receptor antagonists (ARBs) as compared to those without albuminuria. Moreover, they were more likely to be smokers and to show proliferative retinopathy.

We have also compared clinical features of patients with micro or macroalbuminuria. Those with macroalbuminuria showed an evident worse cardiovascular (CV) risk profile as compared to patients with microalbuminuria (Additional file 2: Table S1).

To investigate the differences in the kidney dysfunction progression between patients with low eGFR and normoalbuminuria or albuminuria, a subgroup of 582 patients, whose clinical features are showed in Table 2 and Additional file 2: Table S2, was followed up over a 4-year period. One hundred and twenty five patients (21.5%) showed a loss of eGFR > 30%. The proportion of patients reaching the renal outcome was significantly higher among those with baseline albuminuria as compared to patients with normoalbuminuria (33.6% vs 10.5%, albuminuria vs normoalbuminuria, respectively P < 0.0001). The slope of GFR decline was of − 0.5 (− 2.2; 1.5) mL/min/1.73 m^2^/year for the whole sample. Patients with normoalbuminuria showed a stable kidney function along the follow-up period [i.e. + 0.4 (− 1.2; 3.2) mL/min/1.73 m^2^/year] while those with albuminuria had a significant progression of kidney disease [− 1.3 (− 3.2; 0.3) mL/min/1.73 m^2^/year]. The main baseline clinical features of the above study population grouped by renal outcome at 4-year follow-up are summarized in Table 3. Patients who went on to develop renal outcome showed a poor glycemic control and higher levels of triglycerides and serum uric acid as compared to patients who did not. Furthermore, they had higher levels of SBP despite a greater prevalence of antihypertensive treatment.

**Table 2:** Baseline clinical characteristics of 582 type 1 diabetes mellitus patients with low eGFR and with 4 year follow-up, overall and on the basis of albuminuria

	Overall	Normoalbuminuria	Albuminuria	P
	n = 582	n = 305	n = 277	
Male sex	262 (45.0%)	99 (32.5%)	163 (58.8%)	< 0.001
Age (years)	63 ± 13	65 ± 12	60 ± 13	< 0.001
Duration of diabetes (years)	27 ± 13	26 ± 14	27 ± 12	0.385
BMI (kg/m^2^)	27 ± 5	26 ± 4	27 ± 6	0.085
Serum creatinine (mg/dL)	1.55 ± 0.90	1.31 ± 0.40	1.81 ± 1.19	< 0.001
eGFR (mL/min/1.73 m^2^)	47 ± 12	50 ± 10	43 ± 13	< 0.001
eGFR (mL/min/1.73 m^2^)	50 (39–56)	52 (45–57)	46 (35–54)	< 0.001
Serum uric acid (mg/dL)	5.6 ± 1.6	5.1 ± 1.4	6.1 ± 1.6	< 0.001
Serum uric acid in the top quintile	65 (18.2%)	20 (11.4%)	45 (24.7%)	0.001
HbA1c (%)	8.1 ± 1.5	8.0 ± 1.3	8.3 ± 1.6	0.036
HbA1c (mmol/mol)	65 ± 16.4	64 ± 14.2	67 ± 17.5	0.036
HbA1c ≥ 7%	453 (78.5%)	234 (77.2%)	219 (79.9%)	0.431
HbA1c ≥ 54 mmol/mol	453 (78.5%)	234 (77.2%)	219 (79.9%)	0.431
Total cholesterol (mg/dL)	196 ± 40	197 ± 37	195 ± 43	0.677
Triglycerides (mg/dL)	121 ± 81	110 ± 82	133 ± 79	0.002
Triglycerides ≥ 150 mg/dL	118 (22.1%)	46 (16.6%)	72 (28.0%)	0.002
HDL (mg/dL)	59 ± 19	62 ± 19	57 ± 18	0.004
HDL < 40 M < 50 F mg/dL	105 (19.7%)	54 (19.5%)	51 (20.0%)	0.884
LDL (mg/dL)	113 ± 33	113 ± 31	112 ± 35	0.636
LDL ≥ 100 mg/dL	349 (66.0%)	188 (68.1%)	161 (63.6%)	0.278
Systolic BP (mmHg)	139 ± 19	137 ± 19	141 ± 19	0.032
Diastolic BP (mmHg)	77 ± 9	77 ± 9	78 ± 10	0.204
Blood pressure ≥ 140/85 mmHg	276 (59.2%)	138 (56.1%)	138 (62.7%)	0.146
Non-proliferative retinopathy	123 (21.1%)	64 (21.0%)	59 (21.3%)	0.926
Proliferative retinopathy	68 (11.7%)	24 (7.9%)	44 (15.9%)	0.003
Smokers	46 (18.8%)	20 (14.5%)	26 (24.3%)	0.053
Lipid-lowering treatment	253 (43.5%)	124 (40.7%)	129 (46.6%)	0.151
Treatment with statins	238 (40.9%)	115 (37.7%)	123 (44.4%)	0.101
Treatment with fibrates	8 (1.4%)	5 (1.6%)	3 (1.1%)	0.568
Antihypertensive treatment	421 (72.3%)	199 (65.2%)	222 (80.1%)	< 0.001
Treatment with ACE-Is/ARBs	372 (63.9%)	171 (56.1%)	201 (72.6%)	< 0.001
Aspirin	139 (23.9%)	67 (22.0%)	72 (26.0%)	0.256
Insulin pump	18 (3.1%)	2 (0.7%)	16 (5.8%)	0.003
4-year eGFR reduction > 30%	125 (21.5%)	32 (10.5%)	93 (33.6%)	< 0.001
eGFR at follow-up (mL/min/1.73 m^2^)	47 (33–58)	53 (41–66)	38 (25–49)	< 0.001

Mean ± SD, median (interquartile range) or absolute frequency (percentage). Patients’ baseline missing data: duration of diabetes 18 (3.1%), BMI 108 (18.6%), serum uric acid 225 (38.7%), HbA1c 5 (0.9%), total cholesterol 41 (7.0%), triglycerides 48 (8.2%), HDL 50 (8.6%), LDL 53 (9.1%), blood pressure 116 (19.9%), smokers 337 (57.9%)

*eGFR* estimated glomerular filtration rate, *BMI* body mass index, *HbA1c* glycated haemoglobin, *HDL* high-density lipoprotein cholesterol, *LDL* low-density lipoprotein cholesterol, *ACE-Is* angiotensin converting enzyme-inhibitors, *ARBs* angiotensin II receptor antagonists

**Table 3:** Baseline clinical characteristics of 582 type 1 diabetes mellitus patients reaching the renal end-point (estimated Glomerular filtration rate reduction > 30%)

	4-year eGFR reduction > 30%		P
	No	Yes	
	n = 457	n = 125	
Male sex	197 (43.1%)	65 (52.0%)	0.149
Age (years)	63 ± 13	61 ± 14	0.295
Known duration of diabetes (years)	27 ± 13	27 ± 13	0.726
BMI (kg/m^2^)	26 ± 5	27 ± 6	0.277
Serum creatinine (mg/dL)	1.49 ± 0.86	1.75 ± 1.02	0.501
eGFR (mL/min/1.73 m^2^)	48 ± 11	43 ± 13	< 0.001
Albuminuria	184 (40.3%)	93 (74.4%)	< 0.001
Microalbuminuria	131 (28.7%)	41 (32.8%)	0.001
Macroalbuminuria	53 (11.6%)	52 (41.6%)	< 0.001
Serum uric acid (mg/dL)	5.4 ± 1.5	6.2 ± 1.8	0.004
Serum uric acid in the top gender-specific quintile	40 (14.5%)	25 (30.9%)	0.038
HbA1c (%)	8.1 ± 1.4	8.4 ± 1.5	0.049
HbA1c (mmol/mol)	65 ± 15.3	68 ± 16.4	0.049
HbA1c ≥ 7%	350 (77.3%)	103 (83.1%)	0.129
HbA1c ≥ 53 mmol/mol	350 (77.3%)	103 (83.1%)	0.129
Total cholesterol (mg/dL)	196 ± 38	196 ± 48	0.912
Triglycerides (mg/dL)	114 ± 63	147 ± 125	0.003
Triglycerides ≥ 150 mg/dL	84 (19.9%)	34 (30.4%)	0.054
HDL (mg/dL)	60 ± 19	56 ± 18	0.086
HDL < 40 M < 50 F mg/dL	81 (19.2%)	24 (21.8%)	0.791
LDL (mg/dL)	113 ± 30	112 ± 42	0.964
LDL ≥ 100 mg/dL	280 (66.8%)	69 (62.7%)	0.585
Systolic BP (mmHg)	138 ± 19	143 ± 20	0.040
Diastolic BP (mmHg)	77 ± 9	78 ± 10	0.128
BP ≥ 140/85 mmHg	208 (57.3%)	68 (66.0%)	0.092
Non-proliferative retinopathy	99 (21.7%)	24 (19.2%)	0.850
Proliferative retinopathy	44 (9.6%)	24 (19.2%)	0.008
Smokers	32 (16.8%)	14 (25.9%)	0.060
Lipid-lowering treatment	193 (42.2%)	60 (48.0%)	0.302
Treatment with statins	184 (40.3%)	54 (43.2%)	0.589
Treatment with fibrates	5 (1.1%)	3 (2.4%)	0.308
Antihypertensive treatment	317 (69.4%)	104 (83.2%)	0.010
Treatment with ACE-Is/ARBs	284 (62.1%)	88 (70.4%)	0.226
Aspirin	107 (23.4%)	32 (25.6%)	0.715
Insulin pump	10 (2.2%)	8 (6.4%)	0.050

Mean ± SD or absolute frequency (percentage). Patients’ baseline missing data: duration of diabetes 18 (3.1%), BMI 108 (18.6%), serum uric acid 225 (38.7%), HbA1c 5 (0.9%), total cholesterol 41 (7.0%), triglycerides 48 (8.2%), HDL 50 (8.6%), LDL 53 (9.1%), blood pressure 116 (19.9%), smokers 337 (57.9%)

*eGFR* estimated glomerular filtration rate, *BMI* body mass index, *HbA1c* glycated haemoglobin, *HDL* high-density lipoprotein cholesterol, *LDL* low-density lipoprotein cholesterol, *ACE-Is* angiotensin converting enzyme-inhibitors, *ARBs* angiotensin II receptor antagonists

The P value refers to the effect of each variable on 4-year eGFR reduction > 30% at logistic regression analysis adjusted for baseline eGFR

As expected, they had also a lower eGFR at baseline (43 vs. 48 mL/ min/1.73 m^2^, P < 0.001).

Almost 2/3 of patients who went on to develop eGFR reduction > 30% showed albuminuria at baseline and 19% showed proliferative retinopathy. At the multivariate logistic analysis microalbuminuria, macroalbuminuria and proliferative retinopathy were the only parameters independently associated to eGFR reduction (Table 4).

**Table 4:** Baseline predictors at multivariate analysis for 4-year eGFR reduction > 30%

	Odds ratio	P	Odds ratio	P
Male sex	0.95 (0.55–1.65)	0.861	0.87 (0.49–1.53)	0.620
Age (by 10 years)	0.90 (0.73–1.11)	0.331	0.95 (0.76–1.18)	0.615
eGFR (by 10 mL/min/1.73 m^2^)	0.86 (0.70–1.06)	0.164	0.89 (0.72–1.11)	0.303
Albuminuria	4.09 (2.22–7.56)	< 0.001	–	
Microalbuminuria	–		2.50 (1.26–4.95)	0.009
Macroalbuminuria	–		9.25 (4.44–19.25)	< 0.001
HbA1c (by 1%)	1.10 (0.92–1.31)	0.299	1.06 (0.88–1.27)	0.559
Triglycerides (by 10 mg/dL)	1.02 (0.99–1.05)	0.237	1.02 (0.99–1.06)	0.248
Systolic blood pressure (by 10 mmHg)	1.11 (0.97–1.27)	0.145	1.09 (0.95–1.25)	0.232
Proliferative retinopathy	2.39 (1.22–4.68)	0.011	2.36 (1.18–4.73)	0.016
Antihypertensive treatment	2.19 (0.97–4.92)	0.059	2.14 (0.93–4.92)	0.074

Complete case analysis performed by using a logistic regression model including 427 patients (91 with eGFR reduction > 30%) for which all data were observed

*eGFR* estimated glomerular filtration rate, *HbA1c* glycated haemoglobin

## Discussion

In a large cohort representative of real life clinical practice in Italy, we found that up to 50% of patients with T1DM and CKD show a non albuminuric phenotype. In addition, we demonstrate that the loss of GFR is much greater in T1DM albuminuric CKD as compared with T1DM normoalbuminuric CKD patients.

The prevalence of normoalbuminuric CKD in our population was higher than that in the Finnish Diabetic Nephropathy Study [16] where, of the 502 patients with CKD, 78 (16%) did not have albuminuria. This difference could likely be accounted for by differences in patients’ clinical features and in setting were the patients have been studied. A cross-sectional survey of the UK National Diabetes Audit [9] investigating a large cohort of T1DM patients has recently reported the presence of normoalbuminuria in 54.4% of individuals stage ≥ 3 CKD, a proportion similar to our finding. In a very recent retrospective study by Penno et al. [10], the authors found that, among 29 patients with eGFR < 60 mL/min/1.73 m^2^, 17 (58.6%) had normoalbuminuria. The authors couldn’t find any significant differences for most of clinical variables investigated, very likely because of the small number of patients evaluated. Furthermore, the study conducted by Penno et al. was a cross-sectional, single-centre study while in our study the patients were recruited from more than 200 diabetes clinics in Italy distributed throughout the country and therefore indicative of the clinical practice in the real life condition. The pathophysiology and clinical significance of low GFR levels in patients without albuminuria is to this day unclear. While in patients with T2DM the occurrence of non albuminuric renal impairment has been related to pathogenetic mechanisms such as premature kidney senescence, interstitial fibrosis, ischaemic vascular disease or cholesterol microemboli [3] which may differ from those involved in the development of traditional diabetic glomerulosclerosis, it is currently uncertain whether a similar pathogenetic scenario also applies to T1DM patients with non albuminuric renal dysfunction. Glomerular structural changes typical of diabetic nephropathy, have been reported in T1DM patients with normoalbuminuria and reduced GFR [17]. In these patients, a thickening of the glomerular basal membrane and a greater fractional volume of the glomerulus occupied by mesangium have been found as compared with those who had a normal GFR. Probably the expression of some proteins, such as nephrin, responsible of the integrity of the slit diaphragm, remains unchanged in patients with normoalbuminuric renal insufficiency [18].

When we focused on differences in the kidney dysfunction progression we found that the loss of GFR was greater in albuminuric CKD patients as compared to normoalbuminuric CKD patients. Patients with normoalbuminuria showed a stable kidney function along the follow-up period while those with albuminuria had a significant progression of kidney disease. This finding is in line with the results of Holfiel et al. who described the accelerating effect of albuminuria in the loss of GFR in patients with T1DM and T2DM [19]. Normoalbuminuric renal impairment is a widely described strong risk factor for cardiovascular mortality and morbidity in the general population and in patients with T2DM [20]. Data on this specific issue in patients with T1DM are lacking.

Our study has several limitations as well as strengths. First of all, laboratory variables were not centralized and this could have caused variability especially in the creatinine assay. However, most laboratories around the country currently use the modified Jaffè method, which has good reproducibility. In addition, we have classified our patients using only one measurement of albuminuria. This is in line with other studies [21, 22]. Pugliese et al. [21] have recently reported in a large cohort of subjects with T2DM participating in the renal insufficiency and cardiovascular events (RIACE) Italian Multicentre Study, that a single UAE value, thought to be encumbered with high intra-individual variability, is an accurate predictor of nephropathy stage for clinical and epidemiological purposes. On the other hand, the large number of patients studied and the consistent geographical distribution of the recruiting centers are major strengths of the study, which gives missing information about this issue in Italy. Further limitations merit to be mentioned, i.e. lack of information on: (i) previous cardiovascular events, cancer, infection and hospitalization during the follow-up period, (ii) historical albuminuria data and (iii) duration of smoking habit.

In conclusion, our study shows as the proportion of T1DM patients with normoalbuminuria renal impairment is high (about 50%). These patients have a slower eGFR decline as compared to that observed in patients with albuminuria renal impairment. Whether T1DM patients with albuminuric renal impairment as compared to patients with normoalbuminuric renal impairment need a more intensive renal protection treatment will be clarified by further intervention studies.

## Additional files

**Additional file 1: Fig. S1.** Flow-chart of population.

**Additional file 2: Table S1.** Baseline clinical characteristics of 676 patients with T1DM with low eGFR on the basis of micro- and macro-albuminuria. **Table S2.** Baseline clinical characteristics of 277 patients with DMT1 with low eGFR on the basis of micro- and macro-albuminuria.

## Abbreviations

DKD: diabetic kidney disease
GFR: glomerular filtration rate
T2DM: type 2 diabetes mellitus
T1DM: type 1 diabetes mellitus
CKD: chronic kidney disease
HbA1c: glycated hemoglobin
LDL-c: low-density lipoprotein cholesterol
HDL-c: high density lipoprotein cholesterol
UAE: urinary albumin excretion measurements
BR: background retinopathy
PR: proliferative retinopathy
BP: blood pressure
ORs: odds ratios
ACE-Is: angiotensin converting enzyme inhibitors
ARBs: angiotensin II receptor antagonists
CV: cardiovascular
IDMS: isotope dilution mass spectrometry
