# Supplementary material for: Normoalbuminuric kidney impairment in patients with T1DM: insights from annals initiative
Source: Diabetol Metab Syndr. 2018 Jul 31;10:60. doi: 10.1186/s13098-018-0361-2 (PMC6069993; doi:10.1186/s13098-018-0361-2)
Supplement: Supplementary file 1 — Additional file 1: Fig. S1. Flow-chart of population. [file 13098_2018_361_MOESM1_ESM.doc]

**Title: Normoalbuminuric kidney impairment in patients with T1DM: insights from Annals Initiative**

Authors: Olga Lamacchia, Francesca Viazzi, Paola Fioretto, Antonio Mirijello, Carlo Giorda, Antonio Ceriello, Giuseppina Russo, Piero Guida, Roberto Pontremoli and Salvatore De Cosmo

Address correspondence and reprint requests to: *Olga Lamacchia Unit of Endocrinology and Metabolic Diseases, Department of Surgical and Medical Sciences, University of Foggia, Italy Phone number:+39 0881 732428, Fx number +39 0881 732308; e-mail: olga.lamacchia@unifg.it or *Salvatore De Cosmo, Department of Medical Sciences, Scientific Institute “Casa Sollievo della Sofferenza”, San Giovanni Rotondo (FG), Italy Phone number:+39 0882-410627, Fx number:+39 0882-410627 ; e-mail: [sdecosm@tin.it](mailto:sdecosm@tin.it)

**Additional File 1: Fig. S1. Flow-chart of population**

34,471 patients aged ≥18 years with type 1 diabetes evaluated during the enrollment period in 275 clinics

582 patients from 76 clinics had a 4-year re-evaluation of estimated glomerular filtration rate

18,711 patients from 209 clinics with both estimated glomerular filtration rate (eGFR) and albuminuria evaluations

1,395 patients with eGFR≤60 mL/min/1.73 m^2^ at the last measurement during the enrollment period
