# Supplementary material for: Normoalbuminuric kidney impairment in patients with T1DM: insights from annals initiative
Source: Diabetol Metab Syndr. 2018 Jul 31;10:60. doi: 10.1186/s13098-018-0361-2 (PMC6069993; doi:10.1186/s13098-018-0361-2)
Supplement: Supplementary file 2 — Additional file 2: Table S1. Baseline clinical characteristics of 676 patients with T1DM with low eGFR on the basis of micro- and macro-albuminuria. Table S2. Baseline clinical characteristics of 277 patients with DMT1 with low eGFR on the basis of micro- and macro-albuminuria. [file 13098_2018_361_MOESM2_ESM.doc]

**Title: Normoalbuminuric kidney impairment in patients with T1DM: insights from Annals Initiative**

Authors: Olga Lamacchia, Francesca Viazzi, Paola Fioretto, Antonio Mirijello, Carlo Giorda, Antonio Ceriello, Giuseppina Russo, Piero Guida, Roberto Pontremoli and Salvatore De Cosmo

Address correspondence and reprint requests to: *Olga Lamacchia Unit of Endocrinology and Metabolic Diseases, Department of Surgical and Medical Sciences, University of Foggia, Italy Phone number:+39 0881 732428, Fx number +39 0881 732308; e-mail: olga.lamacchia@unifg.it or *Salvatore De Cosmo, Department of Medical Sciences, Scientific Institute “Casa Sollievo della Sofferenza”, San Giovanni Rotondo (FG), Italy Phone number:+39 0882-410627, Fx number:+39 0882-410627 ; e-mail: [sdecosm@tin.it](mailto:sdecosm@tin.it)

**Additional File 2: Table S1. Baseline clinical characteristics of 676 patients with T1DM with low eGFR on the basis of micro- and macro-albuminuria.**

|  | **Micro** | **Macro** |  |
| --- | --- | --- | --- |
|  | **n=395** | **n=281** | **p** |
| Male sex | 219 (55.4%) | 183 (65.1%) | 0.012 |
| Age (years) | 63±14 | 56±14 | <0.001 |
| Duration of diabetes (years) | 27±13 | 26±12 | 0.171 |
| BMI (Kg/m^2^) | 26.8±5.1 | 26.1±4.7 | 0.088 |
| Serum creatinine (mg/dL) | 1.60±0.79 | 2.03±1.16 | <0.001 |
| eGFR (mL/min/1.73 m^2^) | 45±12 | 40±14 | <0.001 |
| Serum uric acid (mg/dL) | 5.8±1.6 | 6.3±1.6 | 0.002 |
| Serum uric acid in the top quintile | 48 (19.2%) | 54 (29.3%) | 0.014 |
| HbA1c (%) | 8.2±1.6 | 8.5±1.7 | 0.011 |
| HbA1c ≥7% | 303 (77.1%) | 227 (82.2%) | 0.107 |
| Total cholesterol (mg/dL) | 194±43 | 204±55 | 0.015 |
| Triglycerides (mg/dL) | 127±87 | 147±91 | 0.006 |
| Triglycerides ≥150 mg/dl | 90 (24.5%) | 89 (34.0%) | 0.010 |
| HDL (mg/dL) | 59±19 | 57±21 | 0.449 |
| HDL <40M <50F mg/dL | 77 (20.8%) | 59 (22.6%) | 0.589 |
| LDL (mg/dL) | 111±33 | 118±43 | 0.016 |
| LDL ≥100 mg/dL | 214 (59.4%) | 169 (65.3%) | 0.143 |
| Systolic BP (mmHg) | 139±19 | 144±22 | 0.008 |
| Diastolic BP (mmHg) | 77±9 | 79±11 | 0.032 |
| Blood Pressure ≥140/85 mmHg | 192 (59.6%) | 152 (65.0%) | 0.202 |
| Non-proliferative retinopathy | 79 (20.0%) | 48 (17.1%) | 0.339 |
| Proliferative retinopathy | 47 (11.9%) | 43 (15.3%) | 0.200 |
| Smokers | 37 (22.8%) | 30 (26.3%) | 0.507 |
| Lipid-lowering treatment | 169 (42.8%) | 141 (50.2%) | 0.058 |
| Treatment with statins | 160 (40.5%) | 132 (47.0%) | 0.095 |
| Treatment with fibrates | 3 (0.8%) | 2 (0.7%) | 0.943 |
| Antihypertensive treatment | 293 (74.2%) | 240 (85.4%) | <0.001 |
| Treatment with ACE-Is/ARBs | 269 (68.1%) | 209 (74.4%) | 0.078 |
| Aspirin | 107 (27.1%) | 74 (26.3%) | 0.827 |
| Insulin pump | 18 (4.6%) | 17 (6.0%) | 0.389 |

Mean±SD or absolute frequency (percentage).

Abbreviations: eGFR, estimated glomerular filtration rate; BMI, body mass index; HbA1c, glycated haemoglobin; HDL, high-density lipoprotein cholesterol; LDL, low-density lipoprotein cholesterol; ACE-Is, angiotensin converting enzyme-inhibitors; ARBs, angiotensin II receptor antagonists.

**Additional File 2: Table S2. Baseline clinical characteristics of 277 patients with DMT1 with low eGFR on the basis of micro- and macro-albuminuria.**

|  | **Micro** | **Macro** |  |
| --- | --- | --- | --- |
|  | **n=172** | **n=105** | **p** |
| Male sex | 98 (57.0%) | 65 (61.9%) | 0.419 |
| Age (years) | 61±13 | 58±13 | 0.034 |
| Duration of diabetes (years) | 27±12 | 27±11 | 0.671 |
| BMI (Kg/m^2^) | 27±6 | 26±5 | 0.243 |
| Serum creatinine (mg/dL) | 1.67±0.93 | 2.03±1.49 | 0.024 |
| eGFR (mL/min/1.73 m^2^) | 44±12 | 41±15 | 0.044 |
| Serum uric acid (mg/dL) | 5.9±1.4 | 6.3±1.7 | 0.087 |
| Serum uric acid in the top gender-specific quintile | 23 (20.7%) | 22 (31.0%) | 0.119 |
| HbA1c (%) | 8.1±1.5 | 8.5±1.7 | 0.026 |
| HbA1c ≥7% | 131 (76.6%) | 88 (85.4%) | 0.080 |
| Total cholesterol (mg/dL) | 192±42 | 200±44 | 0.120 |
| Triglycerides (mg/dL) | 127±71 | 143±89 | 0.121 |
| Triglycerides ≥150 mg/dl | 41 (25.9%) | 31 (31.3%) | 0.352 |
| HDL (mg/dL) | 58±19 | 56±16 | 0.400 |
| HDL <40M <50F mg/dL | 33 (21.0%) | 18 (18.4%) | 0.607 |
| LDL (mg/dL) | 108±33 | 118±37 | 0.038 |
| LDL ≥100 mg/dL | 92 (59.4%) | 69 (70.4%) | 0.076 |
| Systolic BP (mmHg) | 140±20 | 143±18 | 0.314 |
| Diastolic BP (mmHg) | 77±10 | 78±11 | 0.455 |
| Blood Pressure ≥140/85 mmHg | 81 (60.9%) | 57 (65.5%) | 0.489 |
| Non-proliferative retinopathy | 43 (25.0%) | 16 (15.2%) | 0.056 |
| Proliferative retinopathy | 26 (15.1%) | 18 (17.1%) | 0.655 |
| Smokers | 18 (27.7%) | 8 (19.0%) | 0.311 |
| Lipid-lowering treatment | 77 (44.8%) | 52 (49.5%) | 0.442 |
| Treatment with statins | 72 (41.9%) | 51 (48.6%) | 0.276 |
| Treatment with fibrates | 3 (1.7%) | 0 (0.0%) | 0.291 |
| Antihypertensive treatment | 134 (77.9%) | 88 (83.8%) | 0.234 |
| Treatment with ACE-Is/ARBs | 120 (69.8%) | 81 (77.1%) | 0.183 |
| Aspirin | 47 (27.3%) | 25 (23.8%) | 0.518 |
| Insulin pump | 11 (6.4%) | 5 (4.8%) | 0.573 |
| 4-year eGFR reduction >30% | 41 (23.8%) | 52 (49.5%) | <0.001 |

Mean±SD or absolute frequency (percentage). Abbreviations: eGFR, estimated glomerular filtration rate; BMI, body mass index; HbA1c, glycated haemoglobin; HDL‐c, high‐density lipoprotein cholesterol; LDL‐c, low‐density lipoprotein cholesterol; ACE‐Is, angiotensin converting enzyme‐inhibitors; ARBs, angiotensin II receptor antagonists.
